# Data libraries – the missing element for modeling biological systems

**DOI:** 10.1111/febs.15261

**Published:** 2020-03-10

**Authors:** Anastasia Baryshnikova

**Affiliations:** ^1^ Calico Life Sciences LLC South San Francisco CA USA

**Keywords:** curation, databases, librarianship, modeling, omics, systems biology

## Abstract

The primary bottleneck in understanding and modeling biological systems is shifting from data collection to data analysis and integration. This process critically depends on data being available in an organized form, so that they can be accessed, understood, and reused by a broad community of scientists. A proven solution for organizing data is literature curation, which extracts, aggregates, and distributes findings from publications. Here, I describe the benefits of extending curation practices to datasets, especially those that are not deposited in centralized databases. I argue that dataset curation (or ‘data librarianship’ as I suggest we call it) will overcome many barriers in data visibility and reusability and make a unique contribution to integration and modeling.

## Graphical abstract

Data integration critically depends on data being available in an organized form, so that they can be accessed, understood, and reused. A proven solution for organizing data is literature curation, which is instrumental for integrating findings across publications. Here, I argue that systematic curation of datasets (‘data librarianship’) will have an equally beneficial impact on small, medium, and big data that are released but not organized in a useful manner.

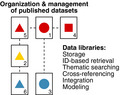

**Abbreviations**
FAIR: findable, accessible, interoperable, and reusable
GEO: Gene Expression Omnibus
GO: Gene Ontology
GTEx: Genotype‐Tissue Expression
HCA: Human Cell Atlas
NLP: natural language processing
OMIM: Online Mendelian Inheritance in Man

## Introduction

Living organisms are exceptionally complex systems and, even after decades of efforts, our understanding of their logical circuitry is fragmentary. The limitations of our knowledge are reflected in the fact that very few biological phenomena have well‐established mathematical models that capture mechanisms in a rigorous yet understandable way and, given known inputs, can accurately predict outputs. The scarcity of such models may seem surprising, considering the wealth of biological data that we collected in recent years and the array of discoveries that such data produced. So why, having large volumes of quality data, aren't we more successful at modeling biological systems?

A good explanation is that building system‐level models that work and make sense is extremely difficult. In most cases, the system of interest (e.g., a dividing cell) has only partially known components (genes, proteins, nutrients, co‐factors) with partially known relationships between them (mutual regulation of abundance, activity, specificity, localization) and partially understood criteria for optimality in any given scenario (growth, division, differentiation, senescence, apoptosis). Modeling such a system requires a long iterative process of analyzing the available data, envisioning how the system might work, translating the vision into mathematical terms, designing additional experiments, and, most importantly, revising the math to be consistent with both old and new information [1].

For the best part of the last two decades, it was fair to assume that the biggest obstacle on our path to modeling was the lack of data and that, once enough data were produced, patterns would emerge and ideas for plausible models would come forward. This reasoning inspired many technological and operational achievements, including the yeast deletion collection [2] and the Human Genome Project [3, 4], that brought rapid progress in automation and parallelization, and increased data production by orders of magnitude. Having acquired tremendous technical capabilities, we realized that the path toward successful modeling is much more tortuous than anticipated. Beyond the data collection challenge, we are now facing an even bigger one – the challenge of combining and integrating different types of data. For example, we now understand that sequencing thousands of human genomes is insufficient to model disease heritability; instead, genotype data must be linked to multiple layers of phenotype data (from clinical records to tissue‐specific molecular biomarkers), as well as environmental and socio‐economic factors. It is still unclear how combinations of these factors affect an individual's progression from health to disease or how, more generally, different types of data fit together to form a full picture of a biological system. What is clear is that learning the principles of this data puzzle will greatly improve our modeling capabilities, and we must direct as much, if not more, effort toward advancing data integration as we did toward scaling data collection.

Here, I suggest that a powerful driver for innovative data integration is great data librarianship, that is, the art and science of dataset management. From my point of view as a researcher and a computational biologist, literature curation has proven incredibly useful for dissemination and widespread reuse of scientific findings. I argue that systematic curation of datasets will have a similar impact on small, medium, and big data that are released but not organized in a useful manner. I propose that a greater focus on data libraries and an explicit support of data librarians will maximize the exposure and reusability of biological data, and, by doing so, lay the foundation for integration and modeling.

## Literature curation has a long successful history

Data integration is a formidable challenge for several reasons [5]. First and foremost, to enable integration, data must be available as widely as possible, while, at the same time, satisfying ethical, legal, and technical requirements. Next, data must be discoverable; that is, a scientist armed with a set of relevant keywords should be able to find the data, even if she was originally unaware of their existence or did not know exactly where to look. Once found, the data must be understandable by those who did not generate it. Finally, having retrieved and understood a diverse family of datasets, scientists must develop new hypotheses on how to integrate them, so that new biological mechanisms, hidden behind the limitations of each individual experiment, can emerge from the union of multiple complementary datasets. Conceiving new integrative ideas is, in and of itself, a heroic endeavor whose challenges and achievements are regularly discussed in the literature [6, 7]. In contrast, a spotlight is long overdue on the equally heroic enterprises that lay the groundwork for integration and make the task easier to approach – the organization and management of datasets.

The scientific community has long promoted efforts to make data globally accessible, discoverable, and understandable [8]. Over 25 years ago, following the release of the first genome sequences and the rise of comprehensive gene catalogs, scientists recognized that, for them to survive the incoming avalanche of new information, they needed to actively gather and organize knowledge about each gene. This need was addressed by widely supporting systematic curation of scientific publications which, at the time, were the main source of biological information. Literature curation, along with database technologies and the Internet, gave us Gene Ontology [9, 10], model organism databases [11], the Online Mendelian Inheritance in Man database [12], and many other resources that became irreplaceable research tools for millions of scientists worldwide. Thanks to literature curators, the reuse of public data and their integration into new biological models became effortless, generating massive returns on the investment in obtaining the data themselves and producing knowledge well beyond the original intent of the experiments [13].

## Data curation is young but profoundly impactful

**Fig. 1:**
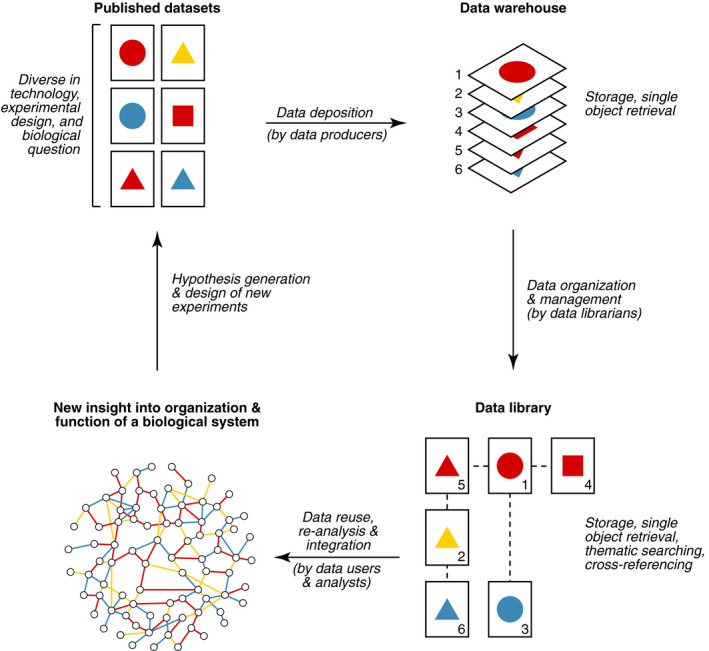
The path from new data to new knowledge lies through data libraries. Unlike data warehouses, which focus primarily on storing and retrieving specific datasets via accession numbers, data libraries actively organize and manage their content, thus enabling advanced searching, improved understanding, and easier reuse and integration of data.

As technology progressed and the influx of data increased, our primary mechanism for gathering information shifted from literature curation to data deposition (Fig. 1), and the role of data producers in making their own data accessible, discoverable, and understandable expanded considerably. For many popular experimental platforms (e.g., microarrays and next‐generation sequencing‐based methods), a successful strategy has been the establishment of central repositories, such as Gene Expression Omnibus (GEO) [14] and ArrayExpress [15], to be populated by direct data submissions from scientists, typically upon publication. Over time, these monumental data hubs have accumulated thousands of independently generated datasets that belong to the same ‘omic’ domain (e.g., transcriptomics), but span numerous organisms, experimental designs, and biological questions, most of which are described in the metadata supplied by the authors.

In contrast to GEO and other submission‐based repositories, data generated by large collaborative consortia, such as the Genotype‐Tissue Expression (GTEx) project [16] and the Human Cell Atlas [17], are often distributed through dedicated portals. These project‐centered repositories host datasets that were obtained, analyzed, and annotated in a standardized way, following a common scientific purpose but harnessing diverse experimental technologies (e.g., genome sequencing, expression profiling, imaging, clinical diagnosis). Organization and management of such datasets requires significant effort as well as specialized skills that neither data producers nor data analysts are typically trained for. The key actors in this process are professional data curators, that is, a group of interdisciplinary individuals with competence in experimental biology, computer science, database administration, information management, and visualization. Depending on their primary area of expertise and circle of responsibility, these specialists may carry different, and sometimes mysterious, titles (e.g., data engineers, curators, wranglers, architects, or stewards), but their vast contributions to scientific research cannot be more obvious: They gather, preserve, and provide access to large‐scale biomedical data, making integrative analyses possible and democratic. In this view (and at the risk of making the problem worse), ‘data librarian’ may be another title added to their roster: Just like traditional librarians, data librarians are custodians and cataloguers of valuable material, and the results of their work provide a critical foundation for education and scholarship (Fig. 1).

Here, the use of a relatively new term (data librarianship) to describe the annotation and organization of datasets may seem redundant and unnecessary. In principle, biocuration is a broad and well‐established concept that applies to scientific information of any kind [13] – why not use it for datasets as well? The reasons, in my opinion, are several. First and foremost, curation takes many different shapes and forms depending on the scope, scale, and projected user base of the curated material. Relative to traditional literature‐based curation [18], the process of organizing datasets and the ways in which scientists interact with its end product are quite different, and a more specific terminology can help emphasizing their unique needs and goals. For datasets, the term ‘library’ seems appropriate as it describes ‘a curated collection of sources of information’ [19], which conveys the difference between ‘source of information’ and the information itself, and highlights the need for a higher‐level organization. Indeed, in a (data) library, sources of information (datasets) are consistently annotated and cross‐referenced to enable navigation, thematic searching, and meta‐analyses that are more complex and more powerful than those using only information (data) from individual sources. The curation of specific data points from each dataset is also important but conceptually closer to literature curation and often accomplished through similar annotation pipelines [20].

Recognizing the distinctive features of data libraries, relative to other repositories of curated data, is also key to appreciating the unique expertise of data librarians, that is, a subgroup of curators who specialize in building and maintaining libraries. Devising and implementing a useful system for organizing a collection of datasets requires a deep and broad understanding of how (and why) these particular data are produced, analyzed, and used. By acquiring such understanding (typically, through close collaboration with a diverse group of experts), a data librarian gains a global ‘data’ view of the field that is inaccessible to many specialists, particularly those that study other, more distant, areas of biology. This global view is an incredible asset to the scientific community as it enables librarians to offer assistance on a variety of data‐related questions (e.g., availability, access, tools, and platforms) to a variety of stakeholders (e.g., researchers, funders, publishers, and policymakers). The ability to advise, consult, or refer users to specific material, similar to what traditional librarians do in a specialized law or medical library, is an underappreciated benefit of systematic dataset curation and would, in my opinion, benefit from a more specific designation.

While being specific with terminology is helpful, it is also not critical and should not hold back the adoption of library practices. The boundaries between data librarianship and other forms of curation are flexible, and some librarian functions can be performed by other data specialists, including curators, producers, and analysts [13]. By adjusting the language, I aim to highlight the unique challenges that dataset curation faces and the unique opportunities that it affords, when done professionally and systematically.

## Data libraries, not warehouses, promote data reuse and integration

The success of GEO, GTEx, and other public initiatives has fueled a culture of open data that promotes free exchange of scientific information in a way that is fast, practical, and beneficial for all parties involved (i.e., data producers, owners, users, and funders) [5]. In most biomedical fields, some degree of data sharing is now expected and also growing thanks to progress in policy, advocacy, and technology [21]. Unfortunately, solutions for organizing existing data have not evolved at the same pace as ideas for producing and sharing new data. By adopting the practice of data deposition, instead of curation, we have effectively moved from data libraries to data warehouses where storage and single object retrieval are greatly prioritized over thematic searching and cross‐referencing (Fig. 1).

While in many ways the change was positive and necessary, it certainly came with compromises. The lack of professional help in preparing a dataset for public release often results in incomplete metadata annotations that may prevent the dataset from being reused in the future. The inconsistency of keyword usage across datasets makes searching unreliable: Just because you could not find a dataset, you cannot be sure that the dataset does not exist. It would be unfair to imply that data libraries, which manage and consistently annotate the data they host, never suffer from similar issues; however, we should expect problems to occur more often if, instead of relying on trained professionals (i.e., data librarians), we delegate annotation tasks to occasional contributors (i.e., data producers) during one of the busiest and most stressful moments of their projects (submission for publication).

## Example of much needed librarianship: medium data

An area where data librarianship is particularly critical is medium‐size datasets. To date, most norms and platforms for data sharing have been developed around mainstream technologies (such as microarrays) or large collaborative projects (such as GTEx), leaving aside data that fit neither of the two categories. Such data may include, for example, metabolomic profiling of an array of cancer cell lines [22], or monitoring the cellular localization of a fluorescent reporter protein in a genome‐wide genetic perturbation screen [23]. Quantitative measurements from such experiments suffer from what can be called a ‘medium data’ problem: They are too big to reside in the main body of a publication, yet not big enough to have an official repository dedicated to their storage.

A common solution to the medium data problem is to release the data, in part or in full, as supplementary material on the journal's website, a general‐purpose digital platform (such as figshare [24], Zenodo [25], and Dryad [26]) or the authors' homepage. These strategies certainly fulfill the minimal requirements for data availability [5], but they are not particularly conducive to integrative data analysis: The datasets spread across multiple locations, custom formats, and varying depths of metadata annotation, all of which increase the activation energy required to find, reuse, and integrate them. Some of these obstacles are greatly reduced by the adoption of data sharing standards [27, 28, 29, 30], which specify format requirements that any dataset, large or small, stored alone, or in a central repository, can conform to. However, understanding and adhering to such standards is not trivial and the effort may seem unjustified for medium data releases that are relatively small and infrequent.

The effective omission of medium‐size data from standards and databases is an underappreciated problem. The dispersion of datasets across platforms and formats prevents rigorous evaluation, leading to very sparse and unreliable estimates of reproducibility. Even more importantly, relative to data produced via massively parallel assays (e.g., RNAseq), medium‐size data (e.g., high content imaging and microscopy) are often produced at lower throughput but higher resolution and greater accuracy, and can therefore capture biological mechanisms from a fundamentally different vantage point. By not recording this information in an accessible, discoverable, and understandable form, we are limiting our chances of reusing and integrating it, and wasting unique opportunities for modeling and innovation.

What can be done to reduce such waste? The strength lies, as is often the case, in the union. While the size of any given dataset may be relatively modest and the benefits of standardizing its format and metadata may seem unclear, the total number of such datasets is incredibly large, and organizing them, based on a shared characteristic, into a data library can be extremely valuable. In this context, organization means (a) extracting relevant experiments from the public domain (relevance may depend on technology, experimental design, or biological question), (b) reformatting and, sometimes, renormalizing the datasets, (c) researching and compiling the appropriate metadata annotations, and (d) distributing the harmonized data in a common and practical format. The organization process should not affect the original data but generate a copy and a permanent link to the source (e.g., the supplementary file, webpage, or figshare object released with the publication). Any manipulation of this copy, such as renaming, restructuring, filtering, or normalizing, should be recorded via self‐contained and version‐controlled code, so as to minimize untraceable human error and provide a historical log of the changes.

It is true that, due to the heterogeneity of medium‐size datasets, their organization almost inevitably involves dataset‐specific operations that are not easy to scale or automate. However, at some steps, information technologies can provide great assistance, making the process easier, faster, or both. For example, Google Dataset Search is a specialized search engine that facilitates discovery of datasets on the Web [31]. The search algorithm relies on data providers describing their datasets using an open standard for structured metadata annotations, Schema.org [32], that captures details about what the data measure, who generated them and how, and what are the terms for reusage. A complement to structured metadata annotations is natural language processing (NLP), a class of machine learning algorithms that can automatically extract relevant information (e.g., experimental and analytical metadata) from large volumes of free‐form documents (e.g., publications). The accuracy of current NLP methods is not yet sufficient to annotate biological datasets without supervision [33, 34, 35]; however, their suggestions can certainly assist human editors in annotating data accurately and efficiently [36, 37, 38, 39].

## The prospects of data librarianship

Fortunately, the need to actively curate, aggregate, and harmonize datasets of all sizes is steadily gaining recognition and motivating a progressive development of data libraries. Databases containing only curated and consistently re‐analyzed datasets are becoming more common (e.g., Expression Atlas [40], gnomAD [41], GenomeCRISPR [42]). Submission‐based data repositories are dedicating part of their resources to create curated data collections (e.g., GEO DataSets [43]) by selecting biologically and statistically comparable experiments among their submissions. Scientific organizations that regularly produce large datasets are developing platforms to share the data in an organized fashion that facilitates integration (e.g., DepMap [44]). Policymakers at national and international levels are taking concrete steps to recognize the pivotal role of data librarians in scientific research and further incentivize their support [45, 46, 47].

Particularly impactful is an interdisciplinary initiative that developed a set of practical recommendations for data producers and publishers to make their data more findable, accessible, interoperable, and reusable (FAIR) [5]. These recommendations, known as the FAIR Data Principles, are designed to facilitate data access for humans and computers by promoting rich metadata annotations, adherence to community standards, search engine optimization, and many other best practices [5]. Thanks to their simple yet powerful philosophy, the FAIR principles have united a diversity of scientific communities under a common goal of increasing the usage of public data and maximizing their impact.

A limitation of the FAIR principles is that implementing them requires exceptional commitment from the data producers as they are the primary source of metadata information and, often, the first data publishers. For data producers, the responsibility of making their data FAIR competes with many other academic responsibilities, such as generating new data, publishing results, and raising funds; however, achieving data FAIRness is not nearly as rewarded as the other goals and is, therefore, difficult to sustain in the long run. Fortunately, this motivational conflict can be resolved in at least two ways, which are not mutually exclusive. First, we can provide data producers with professional FAIR assistance to prevent the burden of data accessibility from resting entirely on their shoulders. Second, we can create powerful incentives that justify the effort of FAIR compliance and make it a global priority.

Our ability to provide professional help with FAIR principles depends on the availability of helpers, that is, data librarians who can work with data producers to organize their datasets, implement the appropriate community standards, and engage with the relevant search engines. Having greater data access benefits all of us; therefore, supporting data librarianship is our shared public responsibility. We must provide centralized funding for data librarianship that is proportional to our spending on data generation. We must allocate human and computational resources to create and maintain data libraries for small, medium, and big data. We must recognize data librarians for their contributions to science and create trajectories for their professional development. Ultimately, we must encourage new ideas on data storage and dissemination in the same way we encourage new technologies and algorithms.

In addition to providing FAIR help through data librarians, we also need to make sure that compliance with FAIR principles is beneficial to data producers and the effort deserves their time. One strategy is to consider measurements of data impact when making decisions about hiring, recognition, and funding. Unfortunately, traditional metrics for publication impact, which measure the number of citations a publication receives, are ill‐suited for capturing the value of the associated datasets [13]. Such value is better reflected by instances of data reuse and re‐analysis, and these account only for a fraction of the citations. Tracking data usage requires new mechanisms, such as a dedicated data citation metric or specialized Web analytics for data access [48, 49]. When implemented, these mechanisms will work at their best with data libraries because, unlike other repositories, they annotate all datasets consistently and provide them with equal opportunity to be discovered. Such balanced visibility enables download and citation rates to reflect true differences in demand, rather than exposure, and encourages data producers to contribute more data.

As discussed above, medium‐size datasets benefit the most from a concerted effort of data librarianship. Yet, among data libraries, those devoted to medium‐size data are particularly underrepresented: Compared to the management of big data, the collation and harmonization of multiple heterogeneous medium‐size datasets is, without a doubt, more time‐ and labor‐intensive, and requires longer‐term vision and planning. Work is in progress to aggregate and jointly examine quantitative data from ~ 15 800 phenotypic screens of the yeast deletion collection [50]. Similar aggregation efforts are ongoing for genome‐wide CRISPR/Cas9 perturbations of mammalian genomes [20, 42], as well as naturally occurring loss‐of‐function variation in human populations [41]. The scientific purpose of each aggregation endeavor will inevitably be different, but the inspiration is one and the same. Through these efforts, independently generated datasets become truly accessible to analysis in a bigger context. Through joint analyses, the reproducibility of biological findings can be fully evaluated, providing a solid foundation for future experiments and hypotheses. Through reliable data, the path to new integrative ideas is more open and clear, and the chances of building good biological models are greatly improved.

## Conflict of interest

AB is a full‐time employee of Calico Life Sciences LLC.
